# The SCID Mouse Model for Identifying Virulence Determinants in *Coxiella burnetii*

**DOI:** 10.3389/fcimb.2017.00025

**Published:** 2017-02-03

**Authors:** Erin J. van Schaik, Elizabeth D. Case, Eric Martinez, Matteo Bonazzi, James E. Samuel

**Affiliations:** ^1^Department of Microbial Pathogenesis and Immunology, College of Medicine, Texas A&M UniversityBryan, TX, USA; ^2^Centre National de la Recherche Scientifique, Formation de Recherche en Évolution 3689, Centre d'études d'agents Pathogènes et Biotechnologies Pour la Santé, Université MontpellierMontpellier, France

**Keywords:** *C. burnetii*, animal model, SCID mouse, transposon mutagenesis, virulence

## Abstract

*Coxiella burnetii* is an intracellular, zoonotic pathogen that is the causative agent of Q fever. Infection most frequently occurs after inhalation of contaminated aerosols, which can lead to acute, self-limiting febrile illness or more serve chronic infections such as hepatitis or endocarditis. Macrophages are the principal target cells during infection where *C. burnetii* resides and replicates within a unique phagolysosome-like compartment, the *Coxiella*-containing vacuole (CCV). The first virulence determinant described as necessary for infection was full-length lipopolysaccarride (LPS); spontaneous rough mutants (phase II) arise after passage in immuno-incompetent hosts. Phase II *C. burnetii* are attenuated in immuno-competent animals, but are fully capable of infecting a variety of host cells *in vitro*. A clonal strain of the Nine Mile isolate (RSA439, clone 4), has a 26 KDa chromosomal deletion that includes LPS biosynthetic genes and is uniquely approved for use in BL2/ABL2 conditions. With the advances of axenic media and genetic tools for *C. burnetii* research, the characterization of novel virulence determinants is ongoing and almost exclusively performed using this attenuated clone. A major problem with predicting essential virulence loci with RSA439 is that, although some cell-autonomous phenotypes can be assessed in tissue culture, no animal model for assessing pathogenesis has been defined. Here we describe the use of SCID mice for predicting virulence factors of *C. burnetii*, in either independent or competitive infections. We propose that this model allows for the identification of mutations that are competent for intracellular replication *in vitro*, but attenuated for growth *in vivo* and predict essential innate immune responses modulated by the pathogen during infection as a central pathogenic strategy.

## Introduction

Q fever is a zoonotic disease caused by the Gram-negative bacterium *Coxiella burnetii*, which most commonly causes asymptomatic or acute febrile illness but can lead to serious chronic infections including endocarditis in humans (van Schaik et al., 2013). The mechanism of transmission is usually inhalation of contaminated aerosols shed from domestic animals (Maurin and Raoult, 1999). The most common animal reservoirs associated with human infection are ruminants, mainly goats and sheep, which contributed to the large outbreak recorded in the Netherlands from 2007 to 2009 (van der Hoek et al., 2012; Brom et al., 2015). After inhalation, *C. burnetii* becomes an intracellular bacterium that invades and proliferates preferentially within (alveolar) macrophages (Khavkin and Tabibzadeh, 1988; Graham et al., 2013). Invasion of macrophages is through a passive mechanism after which the *C. burnetii* vacuole traffics through the default endocytic pathway ultimately to a phagolysosome-like compartment termed the CCV (Baca et al., 1993; Howe et al., 2010; van Schaik et al., 2013). This compartment becomes highly fusogenic and expands concomitant with *C. burnetii* replication (Howe et al., 2003; Coleman et al., 2004) while no apparent bactericidal mechanisms are inhibited. *C. burnetii* manipulates a variety of host pathways to create and maintain its unique intracellular replicative niche including endolysosomal trafficking, secretion, autophagy, and apoptosis (Larson et al., 2015).

It has been particularly challenging to define essential host pathogen interactions with obligate intracellular bacterial pathogens due to the difficulty of culturing them outside of the host. Therefore, understanding virulence determinants of these pathogens has relied on indirect assays. These difficulties have contributed to a lag in progress in defining the pathogenic mechanisms of obligate intracellular bacteria compared to their facultative counterparts. Until recently, the only defined *C. burnetii* virulence factor was full-length lipopolysaccarride (LPS). Serial passage *in vitro* of virulent phase I *C. burnetii* causes a shift to avirulent phase II LPS variants, a phenomenon that is reminiscent of the smooth-to-rough LPS transition common to many enterobacteria (Stoker and Fiset, 1956). This shift from phase I to phase II LPS is most often defined by an irreversible switch characterized by a large chromosomal deletion (Hoover et al., 2002; Beare et al., 2006). Virulence studies using phase I and phase II *C. burnetii* suggest the loss of virulence is due to the binding of C3 to phase II, but not phase I organisms (Moos and Hackstadt, 1987). The observation that phase II *C. burnetii* is less virulent due to the loss of LPS, as a consequence of a permanent 26 KDa chromosomal deletion, led to the exclusion of one clonal derivative from Select Agent designation. Nine Mile II (NMII) RSA439 is approved for use under Biosafety Level 2 (BL2) conditions as compared to the virulent Nine Mile I (NMI) RSA493, which requires Biosafety (Select Agent) Level 3 (BSL3) containment. All *C. burnetii* were strictly obligate intracellular bacteria in the research laboratory until an axenic medium, along with specific growth conditions that allow cultivation of the bacteria outside of host cells, was established (Omsland et al., 2009). As a result of this advance, *C. burnetii* is now a genetically tractable organism, and its virulence can be more directly assessed.

The application of axenic growth conditions led to the development of various genetic tools including stable plasmid vectors allowing the expression of tagged recombinant proteins by *C. burnetii*, a *Himar1* transposon for random mutagenesis, and site-specific mutagenesis strategies (Beare et al., 2011b; Omsland et al., 2011). These innovations have significantly advanced the field and resulted in the identification of several novel virulence factors that are essential for intracellular growth (Beare et al., 2011a, 2014; Carey et al., 2011; Weber et al., 2013; Martinez et al., 2014). The ease of working with the BL2 isolate, coupled with the observation that NMII is comparable to NMI for invasion and replication in a variety of cell types, has led to the description of novel virulence factors using these genetic tools exclusively in the phase II isolate (Howe et al., 2010). Several laboratories have created *Himar1* transposon mutant libraries in NMII as a screening tool to identify novel virulence factors. The use of the *C. burnetii*-adapted *Himar1* transposon was instrumental in characterizing the Type IVB secretion system (T4SS) as an essential virulence factor (Beare et al., 2011a; Carey et al., 2011). Following the observation that the T4SS is required for intracellular replication came descriptions of a growing list of essential secreted effectors, including several of the Cir (Weber et al., 2013), Cvp (Larson et al., 2013, 2015; Martinez et al., 2014), and Ank effectors (Martinez et al., 2014). Other novel *C. burnetii* virulence factors that are T4SS-independent have been identified using transposon mutagenesis, including the invasin, OmpA, which is required for invasion of epithelial cells but not macrophages (Martinez et al., 2014). Therefore, the ability to mutate *C. burnetii* has been instrumental in the identification of genes that are required for uptake and replication in host cells, but continued expansion of these virulence factors will certainly depend on the sensitivity of the screening methods.

Another roadblock in this research field has been the lack of animal models for studying virulence using NMII *C. burnetii* mutants. The *Galleria mellonella* moth model has been applied to identifying essential virulence genes such as the Dot/Icm T4SS, and although this model is an excellent choice for high-throughput studies, it lacks the ability to fully mimic the innate immune responses encountered within a mammalian host (Norville et al., 2014). The immune system of *G. mellonella* consists of the cellular and humoral responses. The cellular response is mediated by hemocytes and involves responses such as phagocytosis, encapsulation, and clotting. Whereas, the humoral response is mediated by opsonins that recognize conserved bacterial products similar to pattern recognition receptors (PRRs) of mammals, antimicrobial peptides, and melanization (Tsai et al., 2016). Therefore, *G. mellonella* has some components that are similar to mammalian innate immune responses. However, the genome is not fully sequenced and there are no methods for creating mutants in *G. mellonella*, and therefore analysis of host-pathogen interactions at the molecular level is not currently possible. In addition, like *Drosophila, G. mellonella* appears to lack NOD-like receptors (NLRs) and therefore is unable to sense certain intracellular threats (Davis et al., 2011). This may account for the similar virulence of wild-type *Legionella pneumophila* and Δ*flaA* in *G. mellonella* as opposed to what is seen in C57BL/6 mice, where activation of Naip5/Ipaf by flagella induces a caspase-1 dependent response. As a consequence, Δ*flaA* mutants replicate to higher numbers than wild-type *L. pneumophila* in a mouse pulmonary model of infection (Harding et al., 2013). With the recent description of the *C. burnetii* effector IcaA, which dampens a non-canonical inflammasome-specific response by inhibiting caspase 11, it will be especially important to employ tractable animal models to validate the contribution of this effector to pathogenesis *in vivo* and discover other mechanisms by which *C. burnetii* modulates host immune responses (Cunha et al., 2015).

Here, we present a SCID mouse model of *C. burnetii* infection that we have used to screen the virulence potential of NMII *Himar1* transposon mutants. The SCID model was used to determine that CirA is required for virulence *in vivo*, which was the first application of this assay (Weber et al., 2016). This report describes the validation of the model and its utility in comparing virulence between NMII strains. An advantage of this model is that it may be used under ABL2 conditions with NMII mutants and therefore does not require creation of site-specific mutants in NMI to test *in vivo* phenotypes. Our data demonstrate that intra-peritoneal (IP) infections in SCID mice are dose-dependent, resulting in splenomegaly characteristic of NMI infections in wild-type mice. In addition, organisms can be found in the spleens, lungs, and hearts of infected animals, showing the potential to identify dissemination defects. This model can also be used to determine relative fitness of a mutant strain in comparison to wild-type *C. burnetii* via single infection and in competitive infection assays.

## Materials and methods

### Bacterial strains, media, and growth conditions

*Coxiella burnetii* RSA439 NMII (clone 4) was used as the parent strain for this study. All mutants used in this study were created using pKM225 containing the *Himar1* transposon and confirmed using rescue cloning as described (Weber et al., 2013) or plTR-CAT-ColE1-P311-GFP and pUCP19::Himar1C9 and confirmed using single primer colony PCR as described (Beare et al., 2009; Martinez et al., 2014). *Coxiella burnetii* wild-type and mutant strains were grown in ACCM-2 at pH 4.75 from Sunrise Science Products (San Diego, CA, USA) for 7 days at 37°C with 5% CO_2_ and 2.5% O_2_. Cultures were then centrifuged at 15,000 × g for 20 min and re-suspended in PBS pH 7.4. Genome equivalents (GE) for each bacterial stock were determined using quantitative real-time PCR as described below.

### Mouse challenge studies

Both SCID (C.B-17/LcrHsd-*Prkdc*^*scid*^) and C57BL6 (C57BL/6NHsd) mice were purchased from Envigo (Indianapolis, IN, USA) and housed in the TAMHSC animal facility. All animal procedures were done in compliance with Texas A&M University IACUC (AUP#2014-0131 and USAMRMC ACURO (CB-2012-28). Six to eight week old female mice (SCID or C57BL/6) were infected with 10^5^–10^8^ of the NMII strain specified in 100 μL PBS pH 7.4 via IP injection or through intra-tracheal (IT) injection using a Biolite intubation system (Braintree Scientific Inc., Braintree, MA, USA) and MicroSprayer® Aerosolizer (Penn-Century Inc.). Inoculum concentrations were confirmed by serial dilution spot plating as described in Sandoz et al. (2016a) on ACCM-2 agarose with or without chloramphenicol selection. For competitive infections, SCID mice were challenged with a 1:1 GE mixture of NMII and *Himar1* transposon mutant, for a total of 10^6^ bacteria per mouse.

### Mouse tissue collection, processing, and DNA purification

3, 7, 14, or 28 days post infection at necropsy the spleens were removed and weighed to determine splenomegaly (spleen weight/body weight). The lungs and hearts were also removed. All organs were individually added to 1 mL PBS pH 7.4 and homogenized using an Omni (TH) equipped with plastic tips (Kennesaw, GA, USA). After homogenization 50 μL of organ was added to 450 μL tissue lysis buffer (Roche) containing 50 μL of proteinase K and incubated at 55 degrees O/N. The following day 50 uL of 10% SDS (w/v) was added and incubated at room temperature for 1 h. Lysed tissue samples were then processed using Roche High Pure PCR template preparation kit according to manufacturer's recommendations (Indianapolis, IN, USA).

### Quantitative real-time PCR

DNA purified from infected organs was used as template for TaqMan real time PCR using primers and probe for *com1* (CBU_1910) (com1_L1: CGCGTTGTCTTCAAAGAACT; com1_R1: GCGTCGTGGAAAGCATAATA; Probe: 5′FAM-CGGCCAATCGCAATACGCTG-3′TAMRA) with NMII genomic DNA serving as the standard (10^7^–10^3^ copies), or primers and probe of IS1111 (IS1111_L1: GAATCAATAACGTCCTTAACATCA; IS1111_R1: CCAATGAGGATTGTCAACGG; Probe: 5′FAM-TGATGAATGTCACCCACGCTCGCA-3′TAMRA). For competitive infections, primers and probe for Himar1 transposon (Himar1_L1: GAGATCAAGCAGAGGCTGAA; Himar1_R1: CTTGGCCTTGTAGGTGGTCT; Probe: 5′FAM-AGGACGGCGGCCACTACGAC-3′TAMRA) with genomic DNA from a *C. burnetii* Himar1 transposon mutant serving as the standard (10^7^–10^3^ copies) to enumerate mutant bacteria. 20 μL reactions were made up with ABI TaqMan universal PCR mastermix and run on ABI StepOne Plus machine. For competitive infections, genomes were quantified using both the IS1111 (for total bacteria) and *Himar1* (for transposon mutants) probe and primer sets. The competitive index (CI) for each infection was calculated by dividing the ratio of mutant-to-wild type genomes in the output sample by that in the inoculum.

### ELIspot

At 28 days post-infection, spleens were removed from NMII-infected mice or naïve control and placed in a 60 mm culture dish with 3 mL of PBS + 1% BSA on ice. Seventy micron sterile nylon mesh was placed onto the spleens and they were mechanically disrupted using a syringe plunger. The spleen cells were placed through another 70 micron nylon mesh filter into 15 mL conical tubes, which were then filled with 10 mL PBS + 1% BSA and centrifuged at 500 × g for 5 min. Naïve splenocytes were resuspended in 1 mL ACK buffer and incubated on ice for 1 min, followed by addition of 10 mL of PBS + 1% BSA and centrifugation at 500 × g for 5 min. Naïve splenocytes were resuspended in 3 mL PBS + 1% BSA and counted using a hemocytometer and diluted to 1 × 10^6^ cells/mL in RPMI complete (RPMI supplemented with 10% FBS) and incubated with 5 μg/mL formalin fixed NMI (WCVI) for 30 min at 37 degrees 5% CO_2_. CD4+ T cells were isolated from NMII-infected splenocytes using Miltenyl Biotec CD4+ isolation kit according to manufacturer's recommendations (San Diego, CA, USA). Isolated CD4+ cells were counted using a hemocytometer and resuspended at 1 × 10^7^ cells/mL in RPMI complete media. R&D Systems Mouse IFN-γ ELIspot® plates were treated according to manufacturer's recommendations and then 100 μL of CD4 + T cells were added to wells in triplicate and either unstimulated, stimulated with Concavalin A (Sigma) or antigen primed naïve splenocytes, and incubated for 24 h at 37 °C in 5% CO_2_. ELIspot plates were developed according to the manufacturer's recommendations and spots were counted using an Immunospot S5 Micro reader from CTL (Cleveland, OH, USA).

## Results

**Figure 1:**
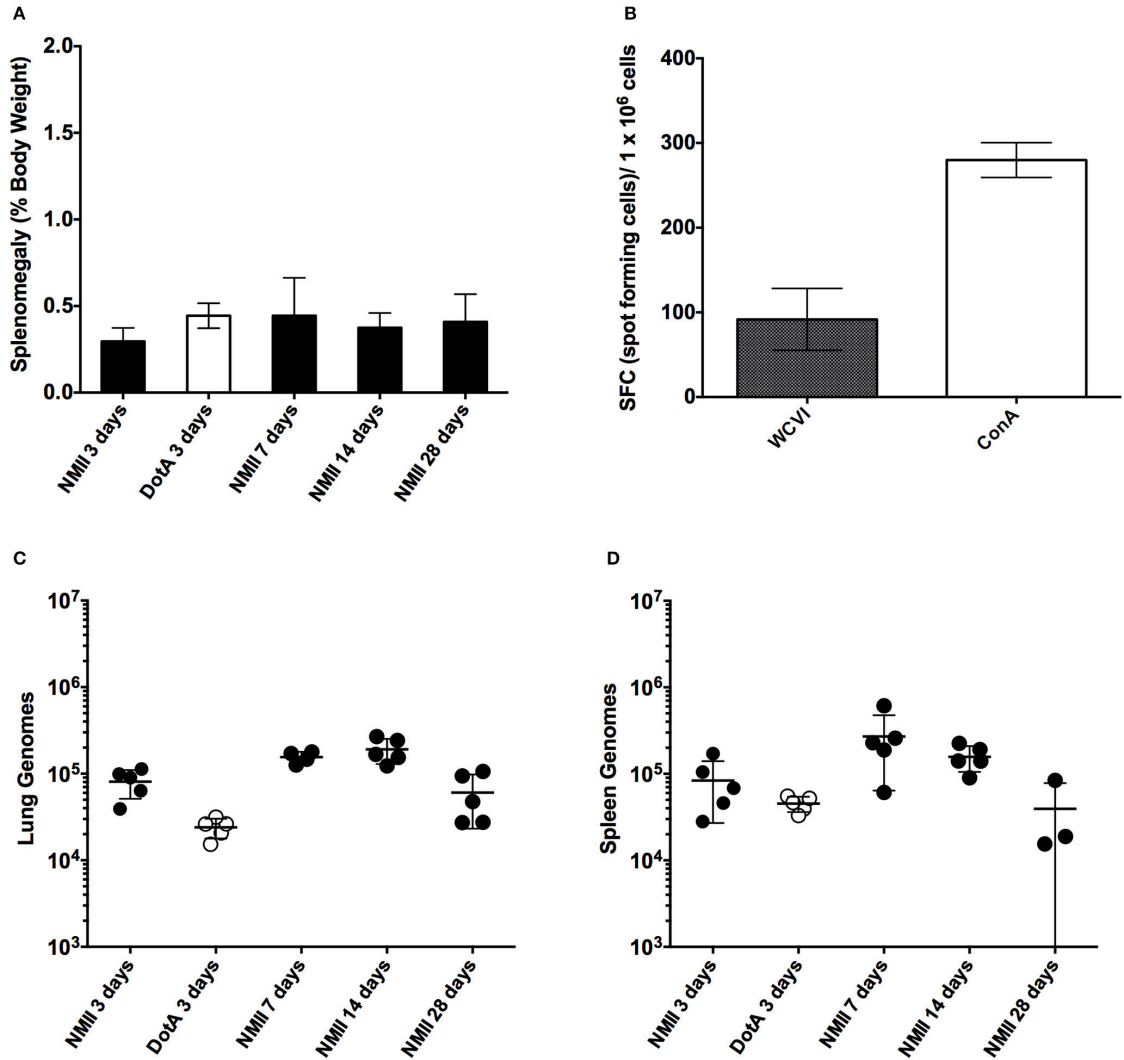
**Time course of intra-peritoneal challenge of C57/BL6 mice with NMII and the specific IFN-γ CD4+ T-cells response. (A)** Splenomegaly calculated as spleen weight as a percentage of total body weight at the time of necropsy on days 3, 7, 14, and 28 after infection with 1 x 10^6^ GE of NMII, or at 3 days after infection with 1 x 10^6^ with *dotA* NMII mutant. **(B)** IFN-γ ELIspot was performed using CD4+ T cells purified from NMII infected spleens at 28 days after infection and stimulated with naïve splenocytes loaded with formalin-fixed NMI (WCVI), or stimulated with concavalin A (ConA). Data are represented as spot forming cells per 1 × 10^6^ cells after deduction of the background spots counted in un-stimulated control wells. **(C)** Genome equivalents calculated using TaqMan real-time PCR with DNA purified from infected lungs from 5 mice on days 3, 7, 14, and 28 days after infection with 1 × 10^6^ GE of NMII or at 3 days after infection for 1 × 10^6^ with *dotA* NMII mutant. **(D)** Genome equivalents calculated using TaqMan real-time PCR with DNA purified from infected spleens from 5 mice on days 3, 7, 14, and 28 days after infection with 1 × 10^6^ GE of NMII or at 3 days after infection for 1 × 10^6^ with *dotA* NMII mutant. For all panels, error bars represent standard deviations from the mean.

Our first objective was to evaluate the utility of an immuno-competent mouse to assess the suitability of predicting relative virulence using NMII and an attenuated derivative of NMI after time course IP challenge using both qPCR to assess genome equivalents and spot plating to determine colonization. Mice were infected with 1 × 10^6^ genome equivalents (GE) IP of either NMII or *Himar1 dotA* mutants and then culled at days 3, 7, 14, and 28 after infection. We saw no significant increase in splenomegaly at any time after infection (Figure 1A). We did, however, see significantly enlarged thymuses in all mice at day 3 (data not shown) and proceeded to evaluate immune response after challenge by IFN-γ ELIspot as a correlate of cell-mediated response 28 days after challenge. We observed that the mice do, in fact, mount a significant antigen specific CD4+ mediated immune response to IP challenge with *C. burnetii* NMII (Figure 1B). Although we did see evidence of steady state GEs in the lungs and spleens at all days post challenge, there were minimal or no viable bacteria as assessed by spot plating. Less than 120 CFU were found in 7 and 14 day spleens (Figures 1C,D and data not shown). We conclude that immune-competent mice are not a feasible model for studying virulence of *C. burnetii* NMII isolates.

**Figure 2:**
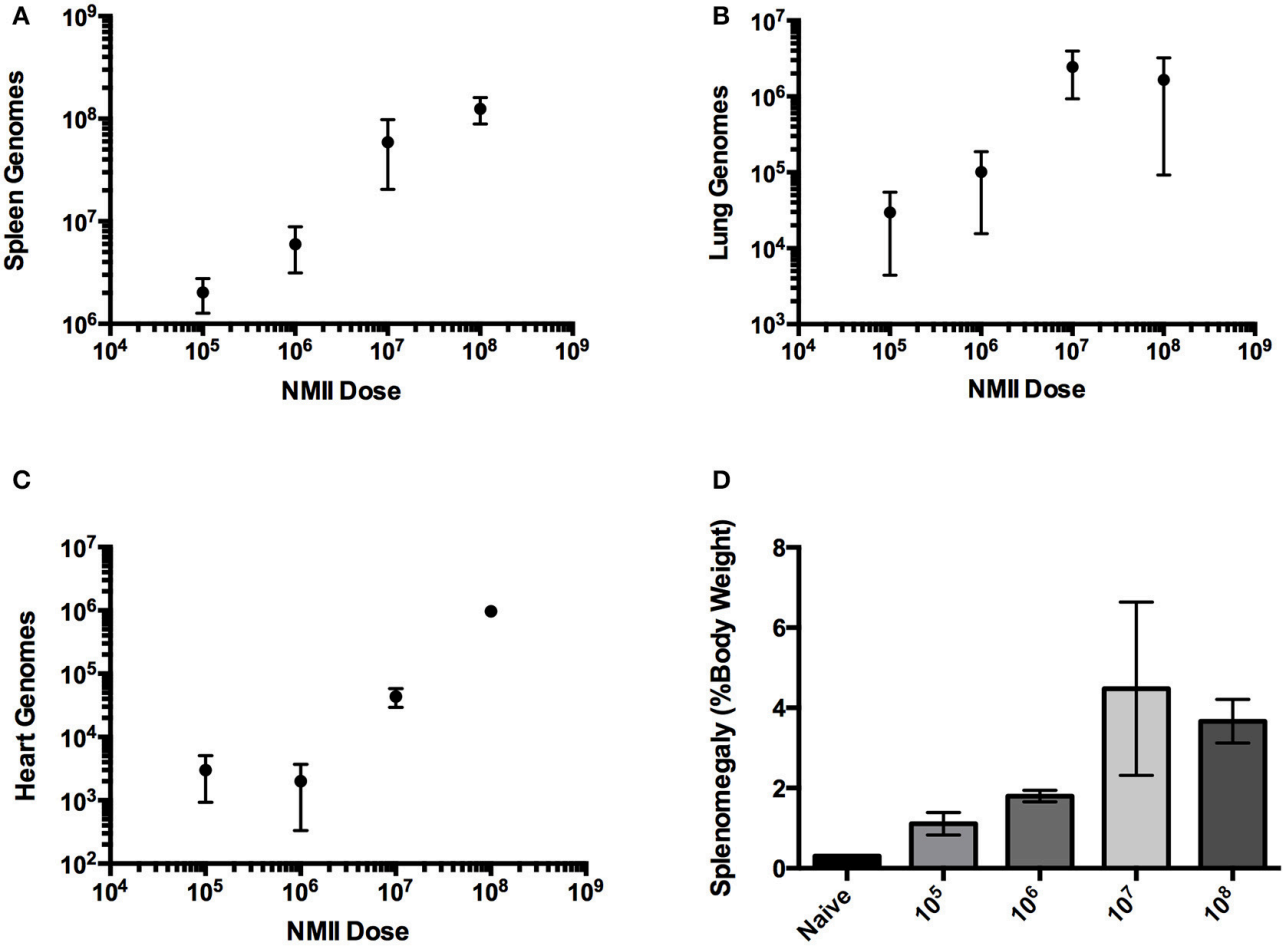
**Infection of SCID mice with NMII is dose-responsive after intra-peritoneal challenge. (A)** Genome equivalents per organ were calculated using TaqMan real-time PCR with DNA purified from infected spleens of 3 mice on days 10 or 14 after intra-peritoneal challenge with 1 × 10^5^ to 1 × 10^8^ GE of NMII. The *R*^2^ (goodness-of-fit) for the linear regression was 0.86 calculated using Prism GraphPad software. **(B)** Genome equivalents per organ were calculated using TaqMan real-time PCR with DNA purified from infected lungs of 3 mice on days 10 or 14 after intra-peritoneal challenge with 1 × 10^5^ to 1 × 10^8^ GE of NMII. **(C)** Genome equivalents per organ were calculated using TaqMan real-time PCR with DNA purified from infected hearts of 3 mice on days 10 or 14 after intra-peritoneal challenge with 1 × 10^5^ to 1 × 10^8^ GE of NMII **(D)** Splenomegaly calculated as spleen weight as a percentage of total body weight at the time of necropsy on days 10 or 14 after infection with 1 × 10^5^ to 1 × 10^8^ GE of NMII.

**Figure 3:**
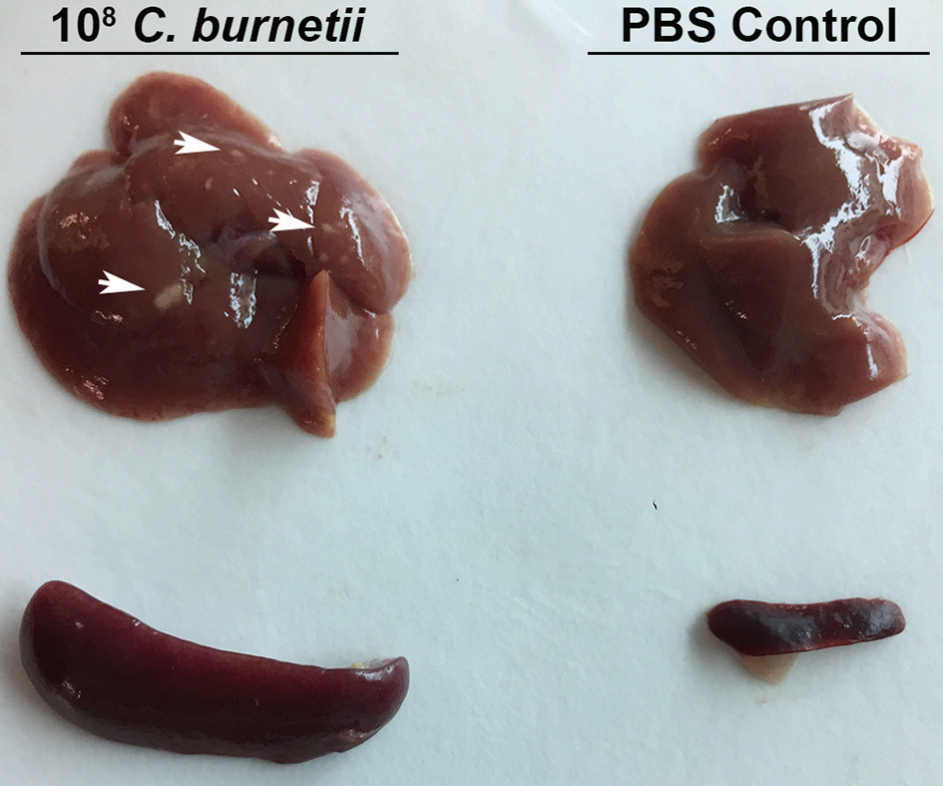
**Gross Pathology of SCID mouse organs after intra-peritoneal challenge**. SCID mice were challenged with 1 × 10^8^ GE or PBS via intra-peritoneal route and sacrificed 10 days after challenge. Spleens and livers were removed from challenged and control mice. Arrows indicate spots of gross pathology on the livers of mice after challenge with NMII.

A dose-response IP challenge was performed in SCID mice with NMII. At 10–14 days post-challenge, genome equivalents were detected in the spleens, lungs, hearts and livers of infected mice (Figures 2A–C and data not shown). At higher doses (10^7^ and 10^8^) gross pathology was observed on the livers, and pericarditis was observed on the hearts of some of the mice (Figure 3 and data not shown). Mice in the 10^8^ challenge group lost more than 20% of their body weight by day 10 and were removed from the study based on animal welfare guidelines. Increasing bacterial loads were found in all organs, and although there was a slight plateau in the 10^8^ group for spleens and lungs, this is probably a result of these mice being removed from the study 4 days before all other doses (Figures 2A–C and data not shown). In addition, there was an increasing trend in splenomegaly from 10^5^ to 10^7^ (Figures 2D, 3). We again speculate that the plateau in splenomegaly observed in the 10^7^ challenge as compared to, the 10^8^ challenge group was a result of these animals being removed from the study prior to day 14. The re-seeding of lungs, liver, and hearts may offer an opportunity to look for dissemination or organ-specific virulence defects.

**Figure 4:**
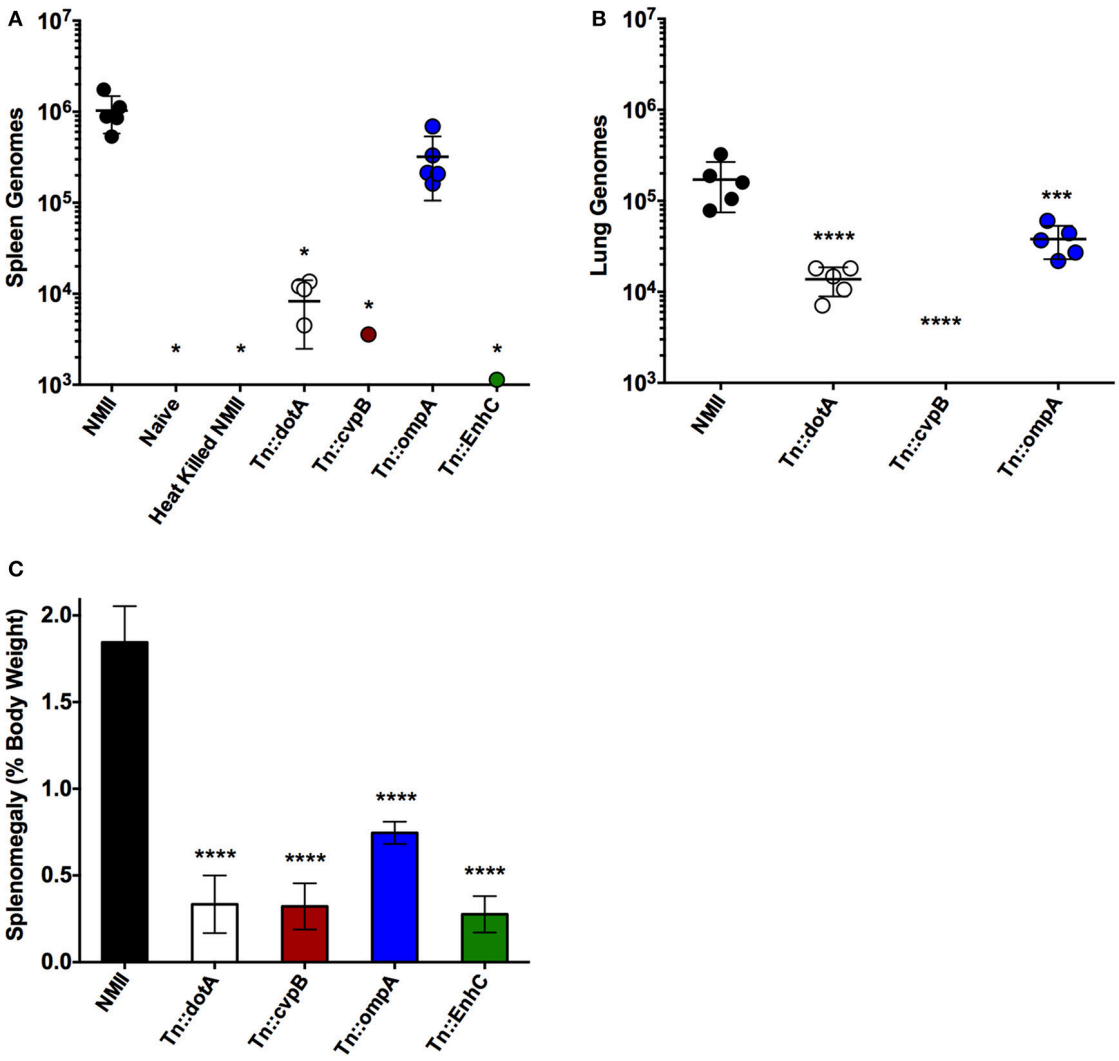
**Intra-peritoneal challenge of SCID mice can be used to test the virulence of NMII mutants. (A)** Genome equivalents calculated using TaqMan real-time PCR with DNA purified from infected spleens of 5 mice per group on day 14 after challenge with 1 × 10^6^ GE equivalents of the strains shown. **(B)** Genome equivalents calculated using TaqMan real-time PCR with DNA purified from infected lungs of 5 mice per group on day 14 after challenge with 1 × 10^6^ GE equivalents of the strains listed in the figure legend. **(C)** Splenomegaly calculated as spleen weight as a percentage of total body weight at the time of necropsy on day 14 after infection with 1 × 10^6^ GE equivalents of the strains listed in the figure legend. For all panels, the data was analyzed using One-way ANOVA followed by the Dunnett's multiple comparisons test against NMII with Prism GraphPad software and significance is displayed using ^*^ for *P* < 0.05, ^***^ for *P* < 0.005, and ^****^ for *P* < 0.0001. Error bars represent standard deviations from the mean.

Based on the dose response studies, subsequent challenges were performed at 1 × 10^6^ GE per mouse, as the splenomegaly observed was comparable to splenomegaly observed after NMI challenge in wild-type mice at similar dose (Andoh et al., 2007). Since steady state GEs were observed in wild-type mice after NMII infection, even though there were few if any culturable *C. burnetii*, the ability to detect heat-killed NMII was determined after 14 days of infection in SCID mice (Figure 4A). The ability of an intracellular replication-deficient mutant *dotA* as well as several other transposon mutants that may have attenuated *in vivo* phenotypes were compared to wild-type NMII (Figure 4A). Splenomegaly was determined and GEs were enumerated in the spleens and lungs of infected mice. There was no evidence of splenomegaly for uninfected or animals challenged with heat-killed NMII, and the GEs were below the limit of detection in the spleens (<1000) (Figures 4A,C). Although there were detectable GEs for *dotA* mutant in both lungs and spleens, they were significantly reduced compared NMII (2 logs and 1 log different, respectively; Figures 4A,B). In addition, there was no detectable splenomegaly for the *dotA* mutant strain (Figure 4C). We then tested *Himar1* mutants that may have attenuated *in vivo* phenotypes including *cvpB* (Martinez et al., 2016), *ompA* (Martinez et al., 2014), and *enhC* (Sandoz et al., 2016b). Of these mutants, *cvpB* and *enhC* were the most attenuated with almost background (naïve/heat killed) levels of GEs in the spleens (*cvpB* and *enhC*) and lungs (*cvpB, EnhC* was not assessed for lung loads), while *ompA* had GEs in both spleens and lungs but the lung load was significantly lower than that of NMII (Figures 4A,B). In addition, there was no evidence of splenomegaly for the *cvpB* and *enhC* mutants, but intermediate splenomegaly for *ompA* (Figure 4C). We confirmed that the *ompA* mutants detected in the spleens were viable by spot plating and consistent with the GE proportions (data not shown). We conclude that the IP challenge model using SCID mice is a feasible model to test the virulence of NMII *Himar1* mutants with two distinct readouts: splenomegaly, as a pathologic correlate, and GEs per organ, as a colonization correlate of disease.

**Figure 5:**
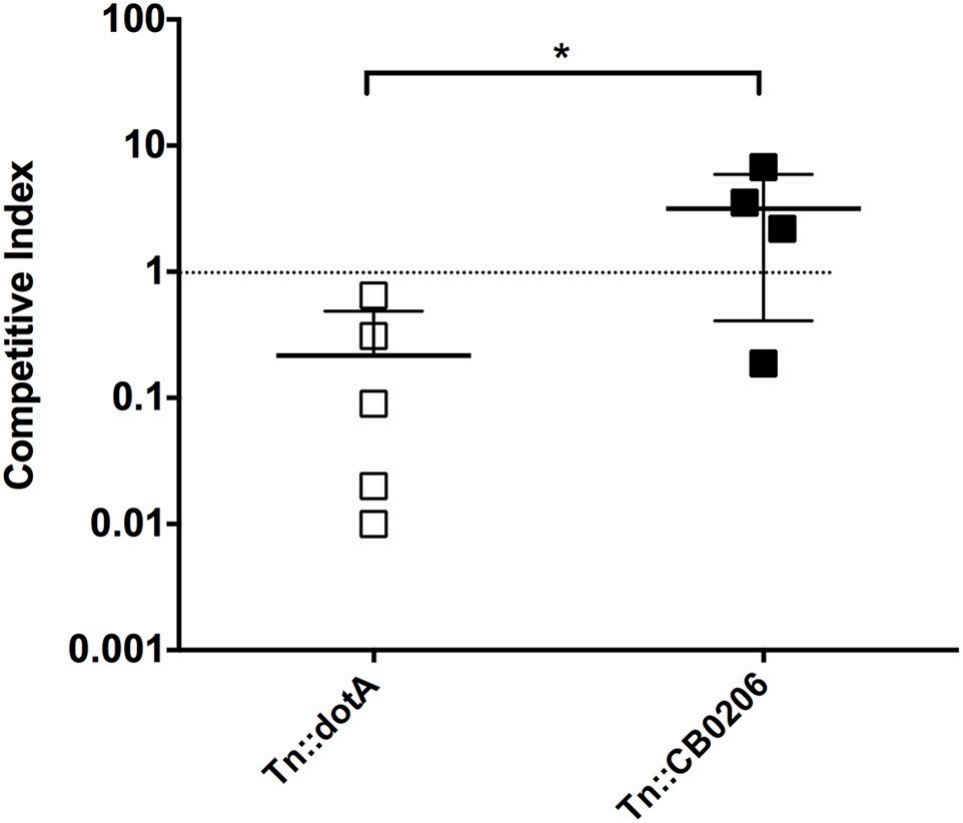
**Fitness of *dotA* and CB0206 Himar1 transposon mutants relative to NMII *Coxiella burnetii***. The relative fitness of *dotA and* CB0206 mutants was compared to wild-type NMII in mixed infections in 5 mice per group where NMII and mutant were inoculated into SCID mice IP in equal proportions. The competitive index (CI) for each infection was calculated by dividing the ratio of mutant-to-wild type genomes in the output sample by that in the inoculum. A CI of 1 indicates the mutant strain has no loss of fitness relative to wild-type bacteria. The data was analyzed using One-way ANOVA followed by the Dunnett's multiple comparisons test against NMII with Prism GraphPad software and significance is displayed using ^*^ for *P* < 0.05. Error bars represent standard deviations from the mean.

We also tested the ability to perform competitive infections with a *Himar1* mutant that is predicted to have comparable virulence to wild-type *C. burnetii* based on the insertion locus, and *dotA*, which is attenuated relative to wild-type (Figure 4A). The competitive index was determined after IP challenge with a 1:1 ratio of mutant to wild-type strains (Figure 5). Consistent with the above results, the *dotA* mutant was indeed found to be less fit than wild-type NMII, with an average competitive index (CI) of 0.216. The other *Himar1* mutant that was tested in the competitive infection assay, Tn::CB0206, was not found to be attenuated, as it successfully competed with wild-type NMII (average CI of 3.16). These results are consistent with our findings that in independent infections, the GE and CFU numbers recovered from spleens infected with the CB0206 mutant were not significantly different from that of wild-type NMII with an average of 2 × 10^7^ and 9 × 10^7^ respectively between mouse groups.

**Figure 6:**
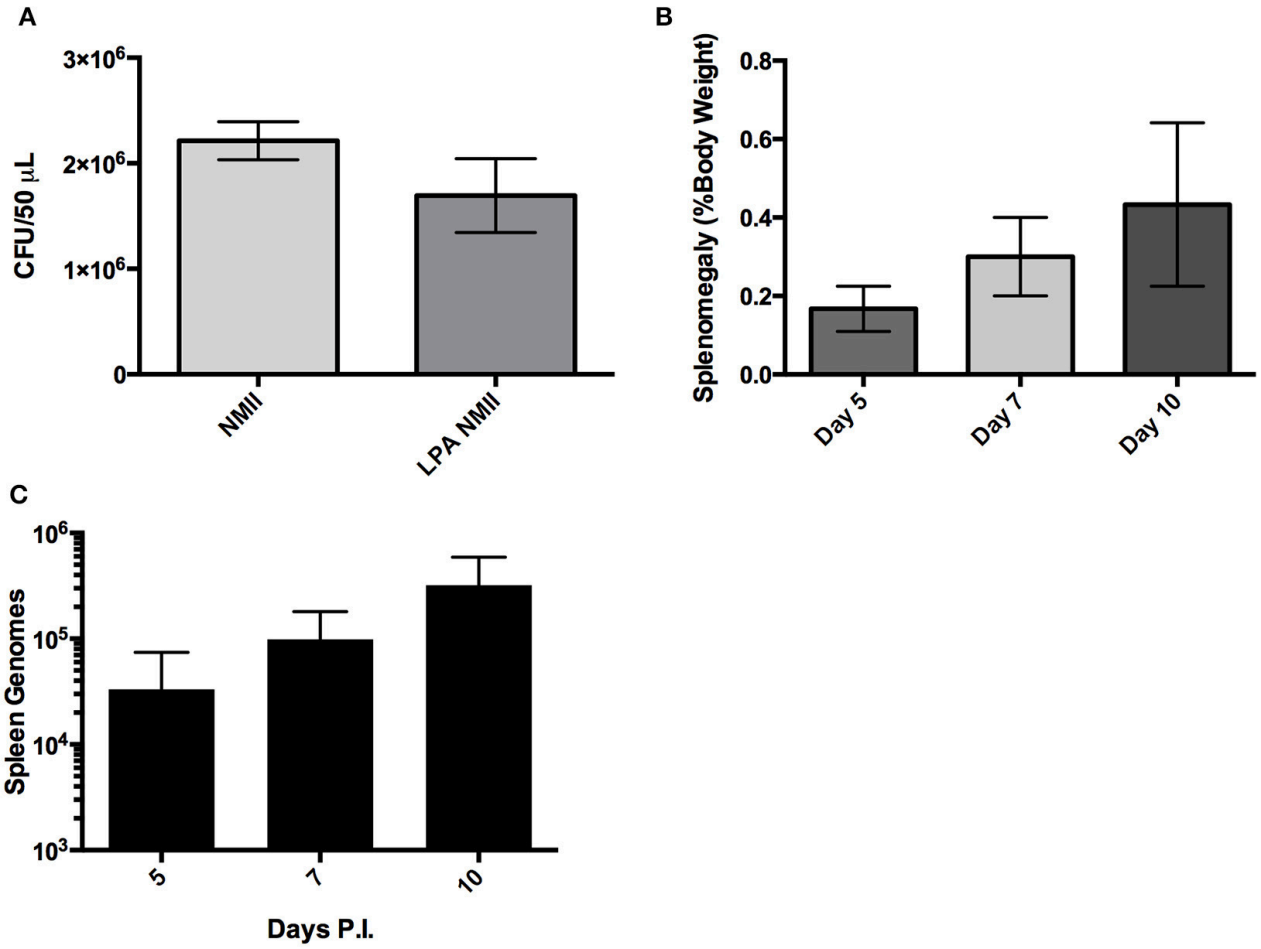
**Large particle aerosolization (LPA) intra-tracheal challenge of SCID mice with NMII. (A)** Colony forming units of NMII present in the inoculum before and after aerosolizaiton with a MicroSprayer® Aerosolizer (Penn-Century Inc.) as determined by spot plating on ACCM-2. **(B)** Splenomegaly expressed as spleen weight as a percentage of total body weight at the time of necropsy on days 5, 7, 10 after intra-tracheal challenge with 1 × 10^6^ GE of NMII. **(C)** Genome equivalents per organ were calculated using TaqMan real-time PCR with DNA purified from infected spleens of 5 mice per group on days 5, 7, and 10 after intra-tracheal challenge with 1 × 10^6^ GE of NMII. For all panels, error bars represent standard deviations from the mean.

Finally, since *C. burnetii* is most commonly transmitted to humans via the respiratory route we evaluated the utility of a potentially more physiologically relevant inoculation route, intra-tracheal (IT) challenge, in the SCID mice. The inoculum was assessed using spot-plating before and after aerosolization with the microsprayer to confirm that the *C. burnetii* inoculated into the lungs remain viable (Figure 6A). Mice were infected (IT) with 1 × 10^6^ GE and then culled at 5, 7, and 10 days after challenge. Splenomegaly was assessed, and although there was a trend from day 3 to 10 of a slight increase in splenomegaly, the difference was not statistically significant, and does not resemble the extensive splenomegaly observed in mice with IP NMI challenge (Figure 6B). In addition, we observed only a modest increase of *C. burnetii* genomes in the lungs and spleen over the time period assessed post-infection (Figure 6C and data not shown). Therefore, although infections lasting greater than 10 days may provide information about virulence, the IT challenge was not a robust model for assessing virulence of NMII isolates.

## Discussion

Here we describe a SCID mouse model for testing *C. burnetii* NMII mutants for virulence. We confirmed that wild-type mice are not a suitable model for predicting the virulence of NMII strains, since no evidence of splenomegaly was observed and only steady state GEs were found in both lungs and spleens. However, the ability to detect NMII GE even 28 days after challenge despite the fact that no viable bacteria were detected based on spot-plating suggest that although NMII is not able to cause productive infections in wild-type mice, it may be able to persist in a viable but non-culturable state. Further evidence to support this hypothesis is that GE from heat killed NMII in SCID mice are not detectable after 14 days, therefore, the detection of GE in wild-type mice even after 28 days suggests that these bacteria are potentially still viable or would have been cleared especially considering wild-type mice mount a specific CD4+ T-cell response (Figure 1B). This observation may have some major implications for the ability of *C. burnetii* to persist within a host for years to decades without causing disease.

SCID mice lack functional B and T cells and therefore adaptive immunity, but do have normal innate immune cells including antigen presenting cells, macrophages, and natural killer cells (Custer et al., 1985). SCID mice have been used to study virulence of intracellular bacterial pathogens including phase I *C. burnetii* (Andoh et al., 2007). It was also recently determined that a quantitative lethal dose 50 (LD_50_) could be determined with NMII in SCID mice using longer experimental periods (Islam et al., 2013) The most physiologically relevant route of challenge would be aerosol, however, the disease progression after IT challenge with NMII in SCID mice was comparably slow and therefore not well-suited to large-scale screening. Furthermore, the increase in splenomegaly and GEs in organs was moderate, indicating a fundamental attenuation in dissemination. It remains to be determined if a longer challenge course (28–48 days) could produce a model of infection that would accurately show dissemination from the lungs to spleen, liver, and heart with splenomegaly, a hallmark of *C. burnetii* challenge in mice. On the other hand, IP challenge of SCID mice was dose-dependent for both bacterial loads in organs and splenomegaly. In addition, higher bacterial loads produced visible pathological changes in both the liver and heart of infected animals. Using a dose of 1 × 10^6^ GE per mouse, we determined that several *Himar1* mutant strains of *C. burnetii* were attenuated for growth in SCID mice. The observation that the T4SS-deficient strain *dotA* was attenuated in the SCID mouse model is consistent with the previous finding that it does not replicate within host cells.

A panel of *Himar1* mutants that have previously published intracellular replication phenotypes and/or defined mechanisms of action were then tested for virulence in the SCID mouse model. Interestingly, the *cvpB* mutant was severely attenuated during SCID mouse infection, yet has no replication defect in tissue culture cells despite its unusual multivacuolar phenotype (Newton et al., 2013; Martinez et al., 2016). CvpB is involved in CCV development by interaction with phosphoinositides and manipulating phosphatidylinsoitol 3-phosphate metabolism to facilitate CCV biogenesis (Martinez et al., 2016). The phenotype in SCID mice was even more severe than what was observed in the *G. mellonella* challenge model, where *cvpB* had a 50% reduction in mortality, confirming that proper CCV biogenesis is essential for *C. burnetii* pathogenesis (Martinez et al., 2016). This supports the hypothesis that the SCID mouse model can be used to identify mutants that do not have intracellular growth defects but are attenuated for virulence.

OmpA is an invasin that is necessary and sufficient for *C. burnetii* to invade non-phagocytic cells (Martinez et al., 2014). Like *cvpB*, it was previously determined that *ompA* mutants had an attenuated phenotype compared to wild-type NMII in *G. mellonella* (Martinez et al., 2014). This phenotype was recapitulated in the SCID mouse model. The GE loads and splenomegaly observed for *ompA* were significantly lower than those observed for NMII, again validating the use of the SCID mouse model to evaluate a range of attenuated virulence phenotypes in NMII. Furthermore, tools unique to *in vivo* infection models, such as FACs analysis could determine what types of cells are infected with *ompA* to further dissect its role in virulence in this animal model.

EnhC was the last potential virulence factor tested in the SCID mouse model of infection. The *Himar1 enhC* mutant was severely attenuated in SCID mice as determined by GE in spleen and lack of splenomegaly. There are several reasons that an *enhC* mutant may be attenuated for growth *in vivo*. First, a previous report has established that a *C. burnetii enhC* mutant had an internalization defect, a phenotype that is also observed for *enhC* mutants in the closely related bacterium *Legionella pneumophila* (Cirillo et al., 2000; Weber et al., 2016). More recently, it was found that EnhC of *L. pneumophila* inhibits the function of SltL transglycosylase by interfering with peptidoglycan degradation, which benefits intracellular replication by limiting the activation of host Nod1 by cell wall components (Liu et al., 2012). Each of these functions could make significant contributions to virulence *in vivo*. In addition, it was recently determined that *enhC* expression is up-regulated during the transition to SCV in *C. burnetii* suggesting it may have a role in cell-remodeling during phase transition (Sandoz et al., 2016b). Any or all of these functions could cause the severe attenuation phenotype we observed in SCID mice. More research is necessary to resolve the function of *C. burnetii* EnhC and its role in pathogenesis.

The ability to define relative fitness for *C. burnetii* mutants by infection of SCID mice allows for an estimation of the fitness cost of a mutation. This is an important step toward testing pools of mutants *in vivo*, which will accelerate the identification of novel virulence factors for *C. burnetii*. This advance, with the advent of a novel Arg selection system for *C. burnetii* that takes advantage of *C. burnetii* auxotrophic status for selection *in vitro* and the Tn7 system for endogenous expression level complementation should allow for the testing of Koch's postulates to define virulence factors (Beare et al., 2011b; Sandoz et al., 2016a).

## Author contributions

Conception and Design of work: Ev, EC, EM, MB, and JS. Drafting work: Ev and EC. Revising critically: Ev, EC, EM, MB, and JS. Final approval: Ev, EC, EM, MB, and JS.

## Funding

This work was supported by DTRA funding grant HDTRA1-13-1-0003 and NIAID NIH A1090142 to JS.

### Conflict of interest statement

The authors declare that the research was conducted in the absence of any commercial or financial relationships that could be construed as a potential conflict of interest.
